# N-Glycosylation Network Construction and Analysis to Modify Glycans on the Spike (S) Glycoprotein of SARS-CoV-2

**DOI:** 10.3389/fbinf.2021.667012

**Published:** 2021-06-08

**Authors:** Sridevi Krishnan, Giri P. Krishnan

**Affiliations:** ^1^ Department of Nutrition, University of California Davis, Davis, CA, United States; ^2^ School of Medicine, University of California San Diego, La Jolla, CA, United States

**Keywords:** N-glycan biosynthesis pathway, SARS-CoV-2, glyco-informatics, computational modeling, glycan heterogeneity

## Abstract

**Background:** The N-glycan structure and composition of the spike (S) protein of SARS-CoV-2 are pertinent to vaccine development and efficacy.

**Methods:** We reconstructed the glycosylation network based on previously published mass spectrometry data using GNAT, a glycosylation network analysis tool. Our compilation of the network tool had 26 glycosyltransferase and glucosidase enzymes and could infer the pathway of glycosylation machinery based on glycans in the virus spike protein. Once the glycan biosynthesis pathway was generated, we simulated the effect of blocking specific enzymes—swainsonine or deoxynojirimycin for blocking mannosidase-II and indolizidine for blocking alpha-1,6-fucosyltransferase—to see how they would affect the biosynthesis network and the glycans that were synthesized.

**Results:** The N-glycan biosynthesis network of SARS-CoV-2 spike protein shows an elaborate enzymatic pathway with several intermediate glycans, along with the ones identified by mass spectrometric studies. Of the 26 enzymes, the following were involved—Man-Ia, MGAT1, MGAT2, MGAT4, MGAT5, B3GalT, B4GalT, Man-II, SiaT, ST3GalI, ST3GalVI, and FucT8. Blocking specific enzymes resulted in a substantially modified glycan profile of SARS-CoV-2.

**Conclusion:** Variations in the final N-glycan profile of the virus, given its site-specific microheterogeneity, are factors in the host response to the infection, vaccines, and antibodies. Heterogeneity in the N-glycan profile of the spike (S) protein and its potential effect on vaccine efficacy or adverse reactions to the vaccines remain unexplored. Here, we provide all the resources we generated—the glycans in the glycoCT xml format and the biosynthesis network for future work.

## Introduction

Glycosylation is a very common and complex post-translational modification process of proteins (Shental-Bechor and Levy, 2008). About 70% of human proteins are likely glycosylated (An et al., 2009). N-glycosylation is one of the main types of glycosylation in humans (Reily et al., 2019). N-glycosylation has been shown to have an impact on protein folding and function in several cases (Parodi, 2000; Mitra et al., 2006). Specifically, the site of glycosylation and the type of the glycan—mannose-rich, hybrid, or complex—likely influence protein function and folding. Virus replication involves the host glycosylation machinery for post-translational modifications. So, it can be useful to understand which enzymes are critical to this process. Bioinformatics tools have been used to determine physiological processes in the field of genetics (Lindblom and Robinson, 2011) and cancer biology (Papaleo et al., 2017) successfully, and such tools are slowly coming into existence in the field of glycobiology (Liu and Neelamegham, 2015).

Given the current SARS-CoV-2 pandemic and the role of the spike (S) glycoprotein in the virus entry and infection of host cells (Ou et al., 2020), we chose to map the enzymatic machinery that is responsible for the spike (S) glycoprotein synthesis. The two novel mRNA vaccines that have shown the highest (> 90%) efficacies both train the immune system to produce antibodies against the spike (S) protein of SARS-CoV-2 (Baden et al., 2020; Jackson et al., 2020). However, the greater the degree to which the spike (S) protein is glycosylated, the greater the chances of vaccine failure (Wang et al., 2020). The high degree of glycosylation of the spike (S) protein also suggests the virus can change quickly, rendering vaccines less effective. Hence, understanding the glycosylation pathway more intricately could help improve drug development and assessment of vaccine efficacy. Once the pathway is understood better, it can determine where modified glycoproteins can be used *in vitro* with immune cells or immunoproteins, or simulation studies using molecular dynamic binding, determining the host response (Grant et al., 14991).

Several SARS-CoV-2 spike protein sequences have been reported (Liu et al., 2020), and the glycosylation profile of the spike (S) protein has also been reported by several independent research groups thus far, using sequencing, mass spectrometry, and imaging tools (Watanabe et al., 2020; Shajahan et al., 2020; Wang et al., 2020). We present a summary of the glycosites in SARS-CoV-2 in Table 1. There are two glycosites in the receptor-binding domain (RBD, which binds with ACE2 protein) between N319 and N541, which are N331 and N343. There is some built-in heterogeneity in the type and level of glycosylation at these glycosites (Shajahan et al., 2020). Most mass-spectrometry–based investigations suggest varying combinations of high mannose, hybrid, and complex glycans, with predominantly complex glycans in the RBD (Watanabe et al., 2020; Shajahan et al., 2020; Zhang et al., 2021; Sanda et al., 2021). A specific study on how this heterogeneity affects the pathogenicity or virulence of the virus has not been done yet. However, with the increase in genetic variants, the amino acid sequences in the spike (S) glycoprotein have also been shown to vary (Xu et al., 2020), and Li et al. evaluated about 80 spike glycoprotein variants, which resulted in 26 glycosite modifications (Li et al., 2020). Deletion of both N331 and N343 sites resulted in severe inability of the virus to infect the host cell, not to mention most glycosylation deletions reduced infectivity.

**TABLE 1:** Glycosylation sites on the SARS-CoV-2 spike (S) protein.

SARS-CoV-2 glycosites	Glycosylated per cryo-EM^a^	Glycosylated per MS data^b^ ^,^ ^c^ ^,^ ^d^ ^,^ ^e^
N17	No	Yes^b^ ^,^ ^c^ ^,^ ^d^/no^e^
N61	Yes	Yes
N74	No	Yes
N122	Yes	Yes
N149	No	Yes
N165	Yes	Yes
N234	Yes	Yes
N282	Yes	Yes
N331	Yes	Yes
N343	Yes	Yes^b^ ^,^ ^c^ ^,^ ^d^/no^e^
N603	Yes	Yes^b^ ^,^ ^c^ ^,^ ^d^/no^e^
N616	Yes	Yes
N657	Yes	Yes
N709	Yes	Yes^b^ ^,^ ^c^ ^,^ ^d^/no^e^
N717	Yes	Yes^b^ ^,^ ^c^ ^,^ ^d^/no^e^
N801	Yes	Yes
N1074	Yes	Yes
N1098	Yes	Yes
N1134	Yes	Yes
N1158	No	Yes
N1173	No	Yes
N1194	No	Yes

a Walls AC et al., Structure, function, and antigenicity of the SARS-CoV-2 spike glycoprotein. Cell, 180: 281–292, 2020.

b Shajahan A et al., Deducing the N- and O-glycosylation profile of the spike protein of novel coronavirus SARS-CoV-2. Glycobiology, 30 (12): 981–988, 2020.

c Watanabe Y et al., Site-specific glycan analysis of the SARS-CoV-2 spike. Science, 369 (6501): 330–333, 2020.

d Zhang Y et al., Site-specific N-glycosylation characterization of recombinant SARS-CoV-2 spike proteins. Molecular and Cellular Proteomics, 20: 100,058, 2021.

e Sanda M et al., N- and O-glycosylation of the SARS-CoV-2 spike protein. Analytical Chemistry, 93 (4): 2003–2009, 2021.

Several groups of researchers have developed bioinformatics tools that are useful in understanding glycosylation, both for glycosite prediction (Pitti et al., 2019) and to predict the biosynthetic pathway based on empirical data from mass spectrometry (Krambeck et al., 2017). GNAT (Liu et al., 2013), which is used in the current manuscript, is a tool that allows for selective identification of glycosylation pathways using the glycan profile of each protein based on specific enzyme rules and constraints. In this work, we examined the effect of blocking two different glycosylation enzymes, to see if we can modify the network of glycans developed as part of the virus spike glycoprotein. Specifically, we chose to simulate blocking mannosidase-II, which would be the effect if swainsonine or deoxynojirimycin were used, and to simulate blocking alpha-1,6-fucosyltransferase, which would be the effect if indolizidine were used. In fact, indolizidine extracts from the *Tylophora* genus of vines have previously been shown to be effective against SARS-CoV (Yang et al., 2010) but are yet to be tested in the current pandemic. Expanding on these blocking studies can help identify the ideal targets to choose that affect the virus replication, since those are dependent on the spike glycoprotein, without affecting the host.

## Methods

*Simulated N-glycan biosynthetic network generation*: We used the list of the most representative N-glycans per glycosite detected by Zhang et al. (obtained from Figure 3 in their manuscript) (Zhang et al., 2021) and the N-glycan profile of SARS-CoV-2 as reported by Shajahan et al. (obtained from Figure 4 in their manuscript) (Shajahan et al., 2020) to generate our primary glycosylation biosynthesis networks. Data from Zhang et al. (Zhang et al., 2021) and Shajahan et al. (Grant et al., 14991) were both obtained from recombinant viral proteins expressed in HEK293 cells, complete mass spectrometry data were available, and their identified glycosites were in agreement, as opposed to data from Sanda et al. (Sanda et al., 2021) that were also from HEK293 cells, but had a different set of glycosites predicted (see Table 1). We also did not use data from Watanabe et al. (Watanabe et al., 2020), since their spike glycoprotein was expressed in FreeStyle 293 cells, in order to keep our data source consistent. We generated the glycoCT xml version of these glycans using the glycanbuilder tool (Ceroni et al., 2008) and verified structures using the glycan chemNIST MS database (Glycan). Our supplemental documents provide all the glycans that were used and generated as part of this analysis in the glycoCT xml format.

*GNAT and inferglycan* *pathway*: As mentioned earlier, we used GNAT (Liu et al., 2013), with the additional enzymes developed by Hou et al. (Hou et al., 2016) to have a functional simulation tool for N-glycan biosynthesis with a total of 26 enzymes [c1galT1, b3galt6, GalNAcT-A, GalT-B, FucTH1, FucTH2, FucTLe, B3GalT, IGNT, ManIb, Man-Ia, SiaT, FucT, MGAT1, GNTE, MGAT2, MANI, Man-II, MGAT3, MGAT4, MGAT5, B4GalT, ST3GalI, ST3GalIV, ST3GalVI, and FUCT7]. The reverse inference algorithm derives the N-glycosylation pathway from a given set of reaction products and possible enzymes. For each enzyme, a set of rules and constraints is provided that defines its action. Given a glycan, it is then possible to identify a set of reactions that led to the production of the glycan, by examining all the enzyme rules with constraints in reverse. This will generate either a network of glycans that are predecessors of the given glycan to Man-9, which is the “parent” glycan for this pathway, or will result in an empty set, i.e., the given glycan has no predecessor leading the way to Man-9. Since we provided all the required enzymes to construct the entire glycosylation pathway, we were able to identify all the paths from Man-9 to all the glycans observed in the mass spectrometry studies. Since there is no good way to determine the linkage information (i.e., structure) from the mass-spectrometry–based composition (Klein and Zaia, 2019), there is likely structural heterogeneity that we failed to account for. This heterogeneity can also affect the pathway that was chosen or the enzyme that was involved in the biosynthesis, which is a limitation of our current approach.

*Simulated inhibition of glycan biosynthesis pathway*: Inhibition of all or parts of the glycosylation pathway is lately being considered a likely chemotherapeutic candidate for cancer (Kurosu, 2018) and Alzheimer’s disease (Haukedal and Freude, 2020), and treatment of the novel coronavirus infection could also be approached in a similar way. There are established natural and synthetic inhibitors for blocking or slowing down the rate of glycosylation biosynthetic reactions (Jeffrey et al., 2015–2017). The blocking of these pathways has been discussed and considered in the past (Wojtowicz et al., 2012; Vasconcelos-Dos-Santos et al., 2015). Tunicamycin, for instance, inhibits UDP-HexNAc, which catalyzes the first step of N-glycosylation (Merlie et al., 1982). It is originally considered an antibiotic and is now considered an anti-tumor agent in cancer (Wu et al., 2018). Furthermore, chloroquine and hydroxychloroquine are glycosylation inhibitors, which have been investigated in their effect against SARS-CoV (Vincent et al., 2005) and SARS-CoV-2 (Kalra et al., 2020), albeit their mechanisms are unclear. We identified the N-glycan biosynthesis pathway using GNAT before choosing common enzyme blocking targets. Based on the pathway we were able to detect for the biosynthesis of these specific glycans, we chose to simulate the blocking of Man-II (as done by swainsonine or deoxynojirimycin) and FucT8 (as done by indolizidine). We generated network graphs with glycans as nodes and enzymes as edges with and without the simulated enzyme inhibition.

## Results

**FIGURE 1:**
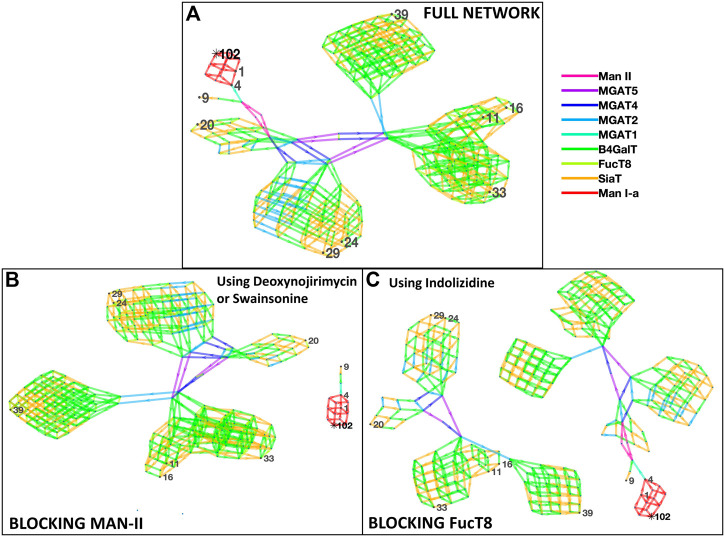
Network of glycans along with the enzymes involved in generating the 10 most abundant glycans as per Zhang et al. [panel **(A)**]. All the 10 glycans (identified by nodes 1, 4, 9, 11, 16, 20, 24, 29, 33, and 39) were traced back to their Man-9 parent glycan (node 102), and the biosynthesis pathway involved the use of nine enzymes as presented in the key to the right, as denoted by the colors. Panels **(B)** and **(C)** display disruption of the network of biosynthesis pathways when using the alpha-mannosidase (Man-II) blocking agent deoxynojirimycin or swainsonine [panel **(B)**] and when using the alpha-1,6-fucosyltransferase (FucT8) agonist indolizidine [panel **(C)**]. In the network generated with enzymes in panels **(B)** and **(C)**, only three of the total 10 glycans are formed.

Figure 1 depicts the network of reactions (edges colored by enzymes) and glycans (nodes, numbered ones were identified by the mass spectrometry data) generated based on glycans from Zhang et al. The glycans detected by mass spectrometric analyses are numbered, and the intermediate ones generated in the biosynthesis pathway are not numbered. Our results suggest the involvement of the following nine enzymes: Man-Ia, Man-II, MGAT2, MGAT3, MGAT4, MGAT5, B4GalT, SiaT, and FucT8, and the network generated 279 glycans in total. In addition to the full network of biosynthetic reactions, Figure 1 also has panels depicting the effect of using chemical inhibitors swainsonine or deoxynojirimycin for blocking Man-II and indolizidine for blocking FucT8. In both cases, only three of the total 10 glycans were formed, thus modifying the glycan profile of the viral protein. In panels B and C, the network depicting three glycans (1, 4, and 9) that result in being traced back to the Man-9 residue (residue #102) will be formed. The other network that is independent of this (not containing residue#102) comprises all the glycans that would not be formed. Table 2 presents the glycans that could and could not be formed when Man-II and FucT8 were blocked. As previously mentioned, these two inhibitors were chosen because they have been established to specifically inhibit glycosylation enzymes.

**TABLE 2:** List of glycans that were formed *vs.* not formed when specific enzymes were blocked when using data from Zhang et al. Glycan numbers indicate nodes in Figure 1.

Formed	Not formed
**Blocking FucT8**	
Glycan:4 HexNAc2Hex5	Glycan:11 hexnac6hex6fuc1neuac1
Glycan:1 HexNAc2Hex6	Glycan:16 hexnac6hex6fuc1neuac2
Glycan:9 HexNAc3Hex6NeuAc1	Glycan:20 hexnac4hex5fuc1neuac2
	Glycan:24 hexnac5hex6fuc1neuac2
	Glycan:29 hexnac5hex6fuc1neuac3
	Glycan:33 hexnac6hex7fuc1neuac2
	Glycan:34 hexnac7hex7fuc1neuac2
**Blocking Man-II**	
Glycan:4 HexNAc2Hex5	Glycan:11 hexnac6hex6fuc1neuac1
Glycan:1 HexNAc2Hex6	Glycan:16 hexnac6hex6fuc1neuac2
Glycan:9 HexNAc3Hex6NeuAc1	Glycan:20 hexnac4hex5fuc1neuac2
	Glycan:24 hexnac5hex6fuc1neuac2
	Glycan:29 hexnac5hex6fuc1neuac3
	Glycan:33 hexnac6hex7fuc1neuac2
	Glycan:39 hexnac7hex7fuc1neuac2

**FIGURE 2:**
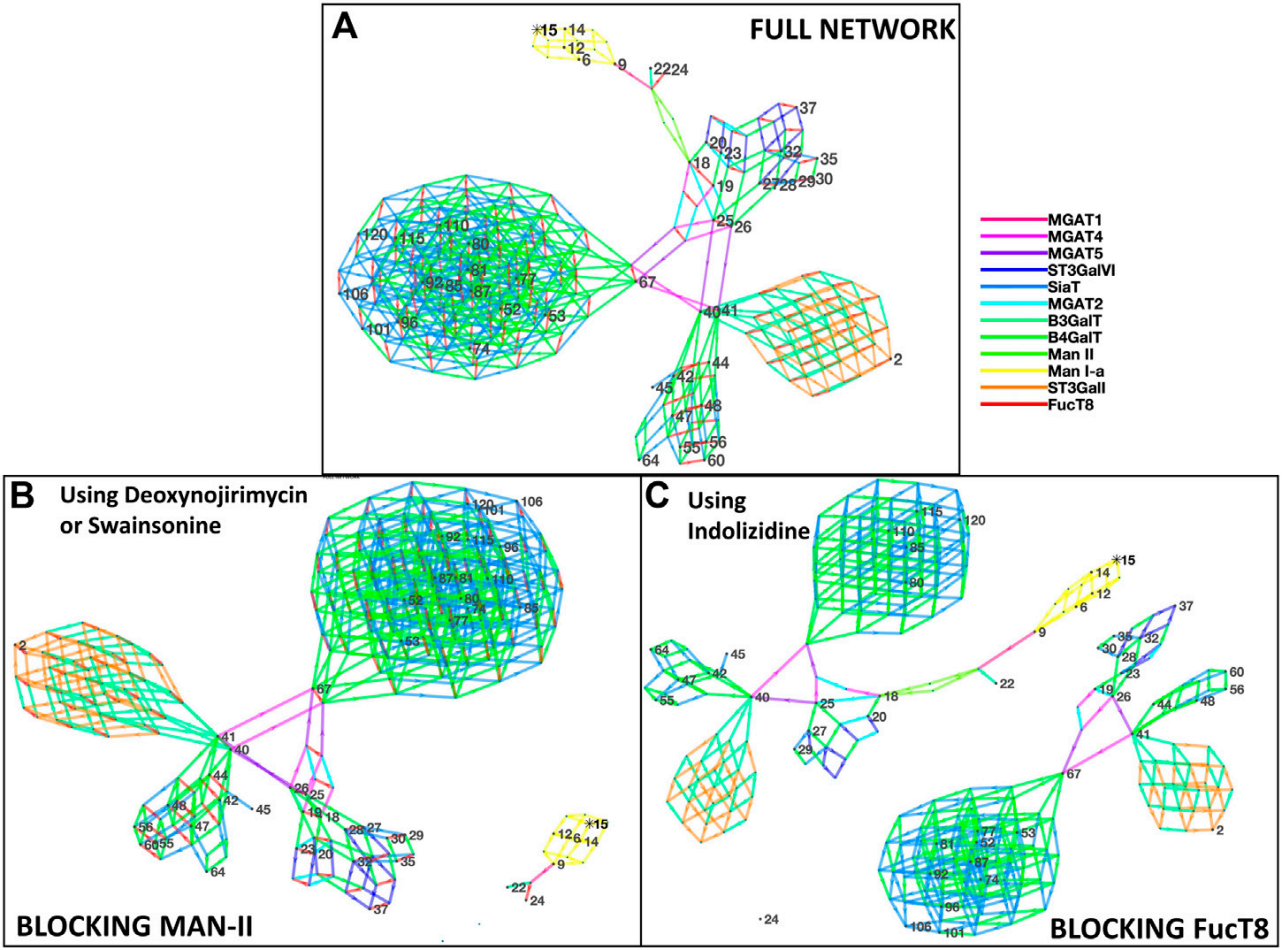
Network of glycans along with the enzymes involved in generating the glycans as per Shajahan et al. [panel **(A)**]. All the 48 glycans (identified by numbered nodes) were traced back to their Man-9 parent glycan (node 15). The colors (explained in the key) depict the different enzymes involved in the biosynthesis pathway. A total of 12 N-glycan biosynthesis enzymes were required to develop this profile of glycans. Panels **(B)** and **(C)** display disruption of the network of glycans generated when using the alpha-mannosidase (Man-II) blocking agent deoxynojirimycin or swainsonine [panel **(B)**] and when using the alpha-1,6-fucosyltransferase (FucT8) agonist indolizidine [panel **(C)**]. In the network generated with enzymes in panel **(B)**, only five glycans are formed, and in panel **(C)**, only 21 of the 48 are formed.

Figure 2 depicts the network of reactions and glycans similar to Figure 1 but is based on glycans from Shajahan et al. Similar to Figure 1, the glycans detected by mass spectrometric analyses are numbered, and the intermediate ones generated in the biosynthesis pathway are not. Here, 12 enzymes were involved—Man-Ia, MGAT1, MGAT2, MGAT4, MGAT5, B3GalT, B4GalT, Man-II, SiaT, ST3GalI, ST3GalVI, and FucT8, and the network generated 292 glycans in total. Similar to Figure 1, blocking of Man-II and FucT8 resulted in a very different glycosylation profile of the overall protein since only the network of glycans that can be traced back to Man-9 (residue #15) will be formed, and the others (networks without residue #15) will not be formed. Table 3 presents the glycans that could and could not be formed when Man-II and FucT8 were blocked.

**TABLE 3:** List of glycans that were formed *vs.* not formed when specific enzymes were blocked when using data from Shajahan et al. Glycan numbers indicate nodes in Figure 2. Red font glycans are on the receptor-binding domain in subunit A of the spike glycoprotein S1.

Formed	Not formed
**Blocking FucT8**	
Glycan:9 hexnac2hex5	Glycan:3 hexnac5hex6fuc1neuac3
Glycan:6 hexnac2hex6	Glycan:19 hexnac3hex3fuc1
Glycan:12 hexnac2hex7	Glycan:23 hexnac3hex4fuc1
Glycan:14 hexnac2hex8	Glycan:24 hexnac3hex5fuc1
Glycan:18 hexnac3hex3	Glycan:26 hexnac4hex3fuc1
Glycan:20 hexnac3hex4	Glycan:28 hexnac4hex4fuc1
Glycan:22 hexnac3hex6	Glycan:30 hexnac4hex4fuc1neuac1
Glycan:25 hexnac4hex3	Glycan:32 hexnac4hex5fuc1
Glycan:27 hexnac4hex4	Glycan:35 hexnac4hex5fuc1neuac1
Glycan:29 hexnac4hex4neuac1	Glycan:37 hexnac4hex5fuc1neuac2
Glycan:29 hexnac4hex5neuac1	Glycan:41 hexnac5hex3fuc1
Glycan:40 hexnac5hex3	Glycan:44 hexnac5hex4fuc1
Glycan:42 hexnac5hex4	Glycan:48 hexnac5hex5fuc1
Glycan:45 hexnac5hex4neuac1	Glycan:52 hexnac5hex5fuc1neuac1
Glycan:47 hexnac5hex5	Glycan:56 hexnac5hex5fuc1neuac1
Glycan:55 hexnac5hex5neuac1	Glycan:60 hexnac5hex6fuc1neuac1
Glycan:64 hexnac5hex6neuac1	Glycan:67 hexnac6hex3fuc1
Glycan:80 hexnac6hex6neuac1	Glycan:53 hexnac6hex5fuc1
Glycan:85 hexnac6hex6neuac2	Glycan:74 hexnac6hex5fuc1neuac2
Glycan:110 hexnac6hex7neuac1	Glycan:77 hexnac6hex6fuc1
Glycan:115 hexnac6hex7neuac2	Glycan:81 hexnac6hex6fuc1neuac1
Glycan:120 hexnac6hex7neuac3	Glycan:87 hexnac6hex7fuc1
	Glycan:92 hexnac6hex7fuc1neuac1
	Glycan:96 hexnac6hex7fuc1neuac2
	Glycan:101 hexnac6hex7fuc1neuac3
	Glycan:106 hexnac6hex7fuc1neuac4
**Blocking Man-II**	
Glycan:9 hexnac2hex5	Glycan:3 hexnac5hex6fuc1neuac3
Glycan:6 hexnac2hex6	Glycan:18 hexnac3hex3
Glycan:12 hexnac2hex7	Glycan:19 hexnac3hex3fuc1
Glycan:14 hexnac2hex8	Glycan:20 hexnac3hex4
Glycan:22 hexnac3hex6	Glycan:23 hexnac3hex4fuc1
Glycan:24 hexnac3hex5Fuc1	Glycan:25 hexnac4hex3
	Glycan:26 hexnac4hex3fuc1
	Glycan:27 hexnac4hex4
	Glycan:28 hexnac4hex4fuc1
	Glycan:30 hexnac4hex4fuc1neuac1
	Glycan:29 hexnac4hex4neuac1
	Glycan:32 hexnac4hex5fuc1
	Glycan:35 hexnac4hex5fuc1neuac1
	Glycan:37 hexnac4hex5fuc1neuac2
	Glycan:29 hexnac4hex5neuac1
	Glycan:40 hexnac5hex3
	Glycan:41 hexnac5hex3fuc1
	Glycan:42 hexnac5hex4
	Glycan:44 hexnac5hex4fuc1
	Glycan:45 hexnac5hex4neuac1
	Glycan:47 hexnac5hex5
	Glycan:48 hexnac5hex5fuc1
	Glycan:52 hexnac5hex5fuc1neuac1
	Glycan:56 hexnac5hex5fuc1neuac1
	Glycan:55 hexnac5hex5neuac1
	Glycan:60 hexnac5hex6fuc1neuac1
	Glycan:64 hexnac5hex6neuac1
	Glycan:67 hexnac6hex3fuc1
	Glycan:53 hexnac6hex5fuc1
	Glycan:74 hexnac6hex5fuc1neuac2
	Glycan:77 hexnac6hex6fuc1
	Glycan:81 hexnac6hex6fuc1neuac1
	Glycan:80 hexnac6hex6neuac1
	Glycan:85 hexnac6hex6neuac2
	Glycan:87 hexnac6hex7fuc1
	Glycan:92 hexnac6hex7fuc1neuac1
	Glycan:96 hexnac6hex7fuc1neuac2
	Glycan:101 hexnac6hex7fuc1neuac3
	Glycan:106 hexnac6hex7fuc1neuac4
	Glycan:110 hexnac6hex7neuac1
	Glycan:115 hexnac6hex7neuac2
	Glycan:120 hexnac6hex7neuac3

The most abundant glycans reported by Zhang et al. (which were used to generate networks presented in Figure 1) were a subset of glycans reported by Shajahan et al. Thus, the network generated by Shajahan et al. was larger and included all the enzymes used by Zhang et al. For the sake of brevity, all information regarding the network is not presented in the manuscript; however, all supplementary information (glycans and high-resolution images of the network and network visualization scripts) can be found at https://github.com/girip/N-glycosylation-network-sars-cov2.git.

## Discussion

Here, we present a preliminary N-glycan biosynthesis pathway of the spike (S) glycoprotein on SARS-CoV-2, which is not available currently for quick examination. We also share the individual glycan structures in a more easily accessible format for future glycoinformatics and molecular dynamics work. Based on our blocking simulation studies, the enzymes Man-II and FucT8 play important roles in the biosynthetic pathway, and without them, the glycans synthesized are altered, changing the glycoprotein profile. The N-glycan biosynthesis pathway is a highly conserved two-step process beginning in the endoplasmic reticulum and ending in the Golgi body (Aebi, 2013). It is now recognized that the host glycosylation process is very relevant to vaccine (Watanabe et al., 2020) and antibody development (Grant et al., 14991). Being able to identify the effect of modified glycans on the spike (S) protein could further aid in the development of vaccines, or in evaluating the efficacy of vaccines by comparing various glycovariant forms of the protein. This first step will pave the way for such future work.

We did not choose to focus only on the glycans in the receptor-binding domain (RBD) in the current report. Instead, we showed that the overall glycan profile is altered dramatically by simulating glycosylation pathway inhibitors. Owing to the site-specific microheterogeneity that all glycoproteins express, which is also true of the SARS-CoV-2 spike protein, it becomes important in subsequent steps to study the effect of this variability on its pathogenicity, with specific focus on the glycans in the RBD. However, the role of these glycans in overall protein folding and dynamics is unclear and still being studied. Several factors determine this microheterogeneity (Varki and et al., 2009), which in turn affects the structure, folding, and dynamics of the protein. So, it becomes important to not focus only on the glycans within the RBD of the spike (S) protein. Researchers have generated molecular dynamic simulations of the SARS-CoV-2 spike protein that represents site-specific microheterogeneity, based on mass spectrometry and imaging studies (Casalino et al., 2020; Chang and Zaia, 2019). Following up on our current approach, it would be possible to generate molecular dynamic simulation studies of the altered spike (S) glycoprotein that is generated by altering the glycosylation machinery.

Various levels of computational modeling of N-glycosylation have been used in the past. Here, we used a pathway construction computational model. Alternatively, simulations that include the kinetics of enzyme activity by including rate equations can be more representative of the physiological process (Krambeck et al., 2009). Using kinetics, it will further be possible to examine the effect of slowing down the rate of glycosylation of the spike (S) protein on overall viral replication (Kumar et al., 2020). Such an approach could shed light on potential pharmacological targets that would slow down both the host and the virus glycosylation pathways. If used appropriately, it may become possible to identify pharmacological targets that would affect the host the least and the virus the most. In addition, the time involved in testing several of these targets to identify ideal ones can be made short by using such network modeling tools before pre-clinical trials. However, this can be very challenging since the mammalian host post-translational glycosylation and its downstream effect on proteins and their functions are still an active area of investigation.

Yet another approach is to use Golgi-compartment representations within the modeling framework to model intra-organelle regional impacts on protein synthesis (Fisher et al., 2019). This could also aid in developing accurate representations of the enzymatic biosynthesis pathway. Alternately, for the current crisis, these approaches could evaluate competitive inhibitor glycans (natural or synthetic) and their effect on viral replication. By generating modified glycoproteins, it is possible to evaluate how they bind to or alter the immune response of the host since the host response to SARS-CoV-2 has recently been shown to be the determining factor in the severity of the manifested infection or for the development of life-threatening adverse complications (Mauro et al., 2020). The added benefit of modeling is to be able to quickly narrow down targets by simulating several at once while also knowing the underlying mechanism, which is not always possible in clinical studies. This makes computational modeling a useful tool in drug and vaccine development efforts.

## Limitations

As mentioned above, there are several other approaches to construct N-glycosylation pathways. In this work, we used pathway construction that does not include dynamics of the glycosylation enzymatic processes. In addition to that, this work being computational is preliminary and requires further computational and experimental/basic/clinical work to identify the effect of simulated outcomes. HEK cells do not mimic lung epithelial or alveolar cells in producing a similar viral titer (Takayama, 2020), so the actual analyses may need to happen by using recombinant virus data from VERO-E6 cells lines, or from lung alveolar cells to match the viral titer or be more accurate. However, the glycosylation pathway enzyme machinery is only missing very few of the components that the mature cells express, making HEK293 cells a good cell line to use (Narimatsu et al., 2019). Another pertinent factor to consider is the wide variability of COVID-19 patient phenotypes contributed by genomic differences (Murray et al., 2020) that could include differences in the glycosylation machinery. We also did not conduct protein dynamic modeling studies, to determine if the altered glycan affects the protein and its downstream binding with mammalian receptors. While factoring in these limitations, we need novel approaches to hasten drug discovery and vaccine development.

## Conclusion

We explored the use of a computational network analysis approach to determine a putative N-glycosylation pathway used by SARS-CoV-2 that results in the spike (S) glycoprotein. Even with emergency use authorization for several vaccines against this virus, mutant strains are being reported frequently (Lauring and Hodcroft, 2021). While the mutations at the genetic level are being reported, there are no efforts to understand how there is heterogeneity in glycosylation and how it affects vaccine efficacy or triggers adverse reactions. We presented here the first step in this process of eventually understanding this aspect better.

## Data Availability

The datasets presented in this study can be found in online repositories. The names of the repository/repositories and accession number(s) can be found in the article/Results section.

## Abbreviations

B3GalT, ganglioside galactosyltransferase; b3galt6, beta-1,3-galactosyltransferase 6;B4GalT, beta-N-acetylglucosaminylglycopeptide beta-1,4-galactosyltransferase; c1galT1, core 1 synthase, glycoprotein-N-acetylgalactosamine 3-beta-galactosyltransferase, 1; FucT, glycoprotein 6-alpha-L-fucosyltransferase; FUCT7, 4-galactosyl-N-acetylglucosaminide 3-alpha-L-fucosyltransferase; FucTH1, type 1 galactoside alpha-(1,2)-fucosyltransferase; FucTH2, type 2 galactoside alpha-(1,2)-fucosyltransferase; FucTLe, 3-galactosyl-N-acetylglucosaminide 4-alpha-L-fucosyltransferase; GalNAcT-A, UDP-GalNAc:polypeptide N-acetylgalactosaminyltransferase A; GalT-B, fucosylgalactoside 3-alpha-galactosyltransferase; GNTE, N-acetyllactosaminide beta-1,3-N-acetylglucosaminyltransferase; IGNT, N-acetyllactosaminide beta-1,6-N-acetylglucosaminyltransferase; MANI, mannosyl-oligosaccharide 1,2-alpha-mannosidase; ManIb, alpha-1,4-glucan-protein synthase; Man-II, mannosyl-oligosaccharide 1,3-1,6-alpha-mannosidase; MGAT1, alpha-1,3-mannosyl-glycoprotein 2-beta-N-acetylglucosaminyltransferase; MGAT2, alpha-1,6-mannosyl-glycoprotein 2-beta-N-acetylglucosaminyltransferase; MGAT3, beta-1,4-mannosyl-glycoprotein 4-beta-N-acetylglucosaminyltransferase; MGAT4, alpha-1,3-mannosyl-glycoprotein 4-beta-N-acetylglucosaminyltransferase; MGAT5, alpha-1,6-mannosyl-glycoprotein 6-beta-N-acetylglucosaminyltransferase; SiaT, N-acetyllactosaminide alpha-2,3-sialyltransferase; ST3GalI, beta-galactoside alpha-2,3-sialyltransferase; ST3GalIV, N-acetyllactosaminide alpha-2,3-sialyltransferase; ST3GalVI, beta-galactoside alpha-(2,6)-sialyltransferase.
